# Unraveling Dysgeusia in SARS-CoV-2 Infection: Clinical and Laboratory Insights from Hospitalized COVID-19 Patients in Romania

**DOI:** 10.3390/pathogens14040300

**Published:** 2025-03-21

**Authors:** Elena Camelia Kouris, Sînziana Irina Mirea, Sigrid Covaci, Monica Luminița Luminos, Victor Daniel Miron

**Affiliations:** 1 Carol Davila University of Medicine and Pharmacy, 050474 Bucharest, Romania; 2 National Institute of Infectious Diseases “Prof. Dr. Matei Balș”, 021105 Bucharest, Romania

**Keywords:** dysgeusia, COVID-19, SARS-CoV-2, Alfa, Beta, Delta

## Abstract

**Introduction:** Dysgeusia has been regarded as a clinical feature associated with SARS-CoV-2 infection since the onset of the pandemic. The initial circulating variants were linked to the presence of dysgeusia; however, with the emergence of the Omicron variant, the incidence of dysgeusia has decreased. The aim of this study was to identify the incidence and characteristics of patients with dysgeusia from the onset of the pandemic to the Omicron variant. **Methods:** We conducted a retrospective study from March 2020 to December 2021, among adult patients hospitalized with COVID-19 in the main infectious diseases hospital in Romania. Clinical and laboratory data were collected and interpreted in relation to the presence or absence of dysgeusia. **Results:** The incidence of dysgeusia was 32.0%, with the majority of cases (44.2%) occurring in patients with the Beta SARS-CoV-2 variant. Dysgeusia has been predominantly observed in mild to moderate cases of the disease. The presence of obesity and hypertension has been shown to be negatively associated with the development of dysgeusia (OR = 0.45, OR = 0.39, respectively). In patients with dysgeusia, inflammatory changes such as lymphopenia were significantly less frequently identified (OR = 0.22, *p* < 0.001), as were increased C-reactive protein (OR = 0.12, *p* < 0.001) and increased interleukin-1 (OR = 0.42, *p* = 0.002), increased fibrinogen (OR = 0.31, *p* < 0.001), and increased ferritin (OR = 0.27, *p* < 0.001) compared to patients without dysgeusia. The incidence of acute respiratory failure was higher in the dysgeusia group (71.2% vs. 28.8%, *p* < 0.001). This was accompanied by a milder management of patients with dysgeusia and a median duration of hospitalization that was two days shorter. **Conclusions**: The presence or absence of dysgeusia in patients with COVID-19 appears to correlate with both the inflammatory response and outcome. In the context of evolving circulating viral variants, which seem to be associated with a lower incidence of dysgeusia, continuous monitoring of patients who develop this disorder remains essential to clarify the pathophysiologic mechanisms involved and to assess the potential of dysgeusia as a predictor of the course of SARS-CoV-2 infection.

## 1. Introduction

The SARS-CoV-2 infection is one of the most widely studied and publicized viral pandemics in history, with a profound impact on both health systems and social life. The consequences of the pandemic have been significant, leading to lasting changes both in medical practice and in the everyday behaviors of the population. The spectrum of clinical manifestations of SARS-CoV-2 infection is highly variable, ranging from asymptomatic forms to severe presentations with multi-organ involvement. Recent research has demonstrated that the virus has the ability to affect multiple organs and systems, contributing to long-term complications, including post-COVID syndrome [1,2,3]. The viral evolution was marked by the emergence of successive variants, each associated with distinct clinical features [4].

While the initial variants were characterized by high mortality and severe disease, in recent years, a decrease in the virulence of SARS-CoV-2 has been observed. This development, coupled with increased population immunity through vaccination and previous infections, has led to a clinical presentation increasingly similar to that of seasonal respiratory viruses [5,6].

Since the advent of the pandemic, dysgeusia, in conjunction with anosmia, has been recognized as a prevalent symptom associated with SARS-CoV-2 infection. The preponderance of cases involving dysgeusia has been observed to coincide with the initial viral variants, namely the Alpha, Beta, and Delta variants; once the Omicron variant emerged, their incidence decreased significantly [7,8,9].

The pathogenetic mechanism of dysgeusia in SARS-CoV-2 infection is not fully elucidated, but several hypotheses have been proposed. These include direct involvement of the olfactory, glossopharyngeal, and facial nerves [10], involvement of the central nervous system in cases with persistent dysgeusia [11], and direct involvement of the taste buds at the lingual level, where ACE2 receptors are abundantly expressed [12]. The simple interaction of the virus with the taste buds could also be a mechanism responsible for the development of this dysfunction [13].

Another hypothesis suggests an important role of proinflammatory mediators, in particular interleukin-6 (IL-6), which could influence the taste pathways through a neuroinflammatory mechanism [14]. Quantitative and qualitative changes in salivary gland secretion may also contribute to altered taste perception [15,16]. Predisposing factors for dysgeusia include age, pre-existing chronic conditions, chronic drug administration, zinc deficiency, and poor oral hygiene [10,15,17].

The aim of this study was to determine the incidence and characteristics of patients hospitalized for SARS-CoV-2 infection and presenting with dysgeusia.

## 2. Methods

We performed a retrospective study based on a previously described protocol [18]. Briefly, all patients hospitalized for SARS-CoV-2 infection on the 10th ward of the National Institute for Infectious Diseases “Prof. Dr. Matei Balș” (NIID) between March 2020 and December 2021 were screened for inclusion in the analysis. The NIID is the largest infectious diseases hospital in Romania and was the main coordinating center for the management of patients with SARS-CoV-2 infection in the country, being dedicated in the first two years of the pandemic only to the care of COVID-19 patients.

The study’s inclusion criteria comprised patients over the age of 18 years who had been diagnosed with SARS-CoV-2 infection via RT-PCR testing of a nasopharyngeal swab, and who exhibited clinical manifestations of varying severity, encompassing mild, moderate, and severe forms of the disease. This selection was undertaken to provide a representative sample for the assessment of the impact of the infection on diverse categories of patients according to the severity of symptoms. However, pediatric patients, asymptomatic patients, and patients who developed critical forms of the disease requiring admission to intensive care units were excluded from the study. Furthermore, patients whose medical records were incomplete or contained inconclusive information on the presence or absence of dysgeusia were excluded, as this symptom was an essential parameter of the analysis.

During the period under analysis, the National Institute for Infectious Diseases “Prof. Dr. Matei Balș” was designated as the first-line medical unit for the management of COVID-19 cases, maintaining this status for two and a half years. Over this time, the hospital has been a reference center for the diagnosis, treatment, and monitoring of SARS-CoV-2-infected patients, providing both specialized medical care and participation in various clinical research initiatives. Patients included in the present study were selected through a rigorous process of consecutive analysis of medical records, according to predetermined inclusion and exclusion criteria. This methodology allowed a systematic and objective approach, ensuring data validity and relevance.

The classification of the clinical forms of COVID-19 has been carried out according to the internationally established criteria set out in the previous protocol [18].

We defined dysgeusia as a taste disorder characterized by altered or distorted taste perception, which can manifest as a reduced ability to taste (hypogeusia), an absence of taste (ageusia), or an unpleasant, distorted taste sensation. In this study, hypogeusia, ageusia, and qualitative taste alterations were collectively documented as dysgeusia.

Data extracted from medical records included relevant epidemiologic and clinical variables: age, sex, background, chronic conditions, circulating viral variants, clinical form of illness, symptoms, time interval between onset of illness and hospitalization, as well as laboratory and imaging investigations performed both on admission and during hospitalization.

Data analysis was conducted using SPSS for Windows (version 25). Categorical variables were compared using the chi-square test, with results expressed as frequencies, percentages, and odds ratios (ORs) accompanied by 95% confidence intervals (95%CIs). For continuous variables, owing to the non-normal distribution of the data, the Mann–Whitney U test was employed, and findings are reported as medians with corresponding interquartile ranges (IQR, 25th to 75th percentiles). Statistical significance was defined by a *p*-value of less than 0.05.

## 3. Results

A total of 1179 records of patients who were hospitalized between March 2020 and December 2021 were analyzed. Of these, 347 patients were eligible and included in the final analysis. The incidence of dysgeusia was 32.0% (*n* = 111). The presence of dysgeusia correlated with female gender (OR = 1.98, 95%CI: 1.24–3.17, *p* = 0.004) and significantly younger age (42 years vs. 52 years, *p* = 0.001) compared to the group of patients without dysgeusia (Table 1).

The presence of dysgeusia did not vary significantly with the circulating variant of SARS-CoV-2 (Table 1) but was more frequent in patients with mild forms of COVID-19.

The majority of patients had at least one chronic condition (66.3%, *n* = 230), but these were significantly rarer in patients with dysgeusia compared to those without dysgeusia (50.5% vs. 73.7%, OR = 0.36, 95%CI: 0.22–0.56). Obesity (34.2% vs. 53.4%, OR = 0.45, 95%CI: 0.28–0.73, *p* = 0.001) and hypertension (27.9% vs. 50.0%, OR = 0.39, 95%CI: 0.24–0.63, *p* < 0.001) were negatively associated with the presence of dysgeusia.

The differences between the two groups in blood tests are highlighted in Table 2. We identified that patients without dysgeusia were associated more frequently with changes in inflammatory parameters. Thus, lymphopenia (45.9% vs. 79.2%, OR = 0.22, 95%CI: 0.13–0.36, *p* < 0.001), increased fibrinogen (45.0% vs. 72.9%, OR = 0.31, 95%CI: 0.19–0.49, *p* < 0.001), increased C-reactive protein (71.2% vs. 95.3%, OR = 0.12, 95%CI: 0.06–0. 25, *p* < 0.001), elevated IL-1 (18.0% vs. 33.9%, OR = 0.42, 95%CI: 0.13–0.36, *p* = 0.002), and elevated ferritin (38.7% vs. 69.9%, OR = 0.27, 95%CI: 0.17–0.43, *p* < 0.001) were significantly more frequent in the group of patients without dysgeusia. Similarly, other biochemical parameters that had more frequently elevated values among those without dysgeusia were LDH (55.9% vs. 75.0%, OR = 0.42, 95%CI: 0.26–0.68, *p* < 0.001), AST (28.8% vs. 50.4%, OR = 0.39, 95%CI: 0. 25–0.65, *p* < 0.001), ALT (39.6% vs. 64.4%, OR = 0.36, 95%CI: 0.23–0.58, *p* < 0.001), amylase (9.9% vs. 23.3%, OR = 0.36, 95%CI: 0.18–0.72, *p* = 0.003), and lipase (10.8% vs. 26.7%, OR = 0.33, 95%CI: 0.17–0.64, *p* = 0.001).

Patients with dysgeusia required a milder management, in which the use of antibiotics (OR = 0.28, 95%CI: 0.15–0.54, *p* < 0.001), antivirals (OR = 0.43, 95%CI: 0.28–0.69, *p* < 0.001), biologic therapies (OR = 0.23, 95%CI: 0.14–0.40, *p* < 0.001), and corticosteroid therapy (OR = 0.08, 95%CI: 0.04–0.16, *p* < 0.001) was significantly less frequent compared to patients without dysgeusia (Table 3).

Acute respiratory failure was more frequent in patients without dysgeusia (OR = 0.16, 95%CI: 0.09–0.27, *p* < 0.001, Table 4). However, the median length of hospitalization was only 2 days longer (*p* = 0.156) in patients without dysgeusia compared to those with dysgeusia.

## 4. Discussion

We carried out a retrospective study among patients hospitalized in the largest infectious diseases hospital in Romania, which for two years was specifically dedicated to the care of COVID-19 patients. Thus, we aimed to identify and analyze patients with dysgeusia in comparison with those without dysgeusia from the onset of the pandemic to the time of the first case of Omicron in Romania [19]. Our study included 347 hospitalized patients with an incidence of dysgeusia of 32%. Our results are comparable with those reported in the meta-analysis by Mutiawati et al. [20], which included 30,901 patients from 101 international studies and estimated an overall incidence of dysgeusia of 36.6%, similar to that identified in our study. In contrast, a study conducted by Ali et al. [21], during the same period of time and including 405 patients diagnosed with COVID-19, reported an incidence of dysgeusia of 48.1%, higher than that observed in our study. This difference could be explained by the higher proportion of patients with mild forms of the disease in Ali et al.’s study (87.4%) compared to our study, where only 8.5% of patients were diagnosed with mild forms.

These data emphasize the variability in the prevalence of dysgeusia depending on the characteristics of the study sample, including the severity of the disease and the patient selection methodology.

Our analysis revealed a predominance of dysgeusia among female patients, with a percentage of 65.8%. This association is further substantiated by the study conducted by Ali et al. [21], which reported that 60.2% of dysgeusia patients were female. With regard to the age distribution, the median age was found to be different between the present study (36 years) and the study by Ali et al. [21] (42 years). Similar results were obtained by Vaira et al. [22], who reported a higher prevalence of dysgeusia among women (62.5%) and a median age of 49.2 years. A study conducted at the beginning of the pandemic in Italy [23] also showed the association of dysgeusia with the female population and younger ages. One pathophysiologic hypothesis suggests that ACE2 receptors, essential for SARS-CoV-2 virus adhesion, are expressed in a higher proportion on the X chromosome, which may explain the increased susceptibility of women to taste perception changes [24]. Although taste alterations are typically more common with advancing age, a number of studies conducted during the pandemic [23,25,26,27] have shown an association of dysgeusia with younger ages and with mild forms of COVID-19. These observations suggest possible differences in immune response and viral receptor expression according to age and sex, warranting further investigation to elucidate the mechanisms involved.

A study conducted by Sheng et al. [27] in Taiwan from January to May 2020 on a total of 217 patients identified an increased incidence of dysgeusia among patients with moderate forms of the disease (29.5%). These results are comparable to those obtained in our study, where 36.1% of patients with dysgeusia had moderate forms of COVID-19. These data support the hypothesis that the presence of dysgeusia might be associated with a more favorable course of SARS-CoV-2 infection. The hospitalization of mild cases in our study was influenced by two key factors. Firstly, during the early phase of the pandemic (March–July 2020), all patients diagnosed with SARS-CoV-2 infection were hospitalized regardless of disease severity, as per national health regulations. Secondly, throughout the pandemic, hospitalization was recommended for elderly patients or those with pre-existing risk factors to enable close monitoring and prevent progression to severe disease. These factors may have influenced the distribution of dysgeusia cases within the hospitalized cohort.

Another significant observation in our study was the low incidence of dysgeusia in obese patients. This has also been reported by Khan et al. [14], who demonstrated that obese patients have a reduced number of olfactory and gustatory receptors, which could explain the lower frequency of dysgeusia in this population. Obesity, on the other hand, is recognized as a major risk factor for severe forms of COVID-19, being associated with a heightened inflammatory response, relative immunosuppression, and increased susceptibility to complications [14,28,29].

It is well known that patients infected with SARS-CoV-2 frequently present with chronic comorbidities such as hypertension, diabetes mellitus, cardiovascular disease, and obesity. A study conducted in Indonesia from January 2020 to September 2021 [30] showed that the incidence of obesity, hypertension, and diabetes mellitus was significantly lower among patients with dysgeusia than in the group of patients without this manifestation. Similar results were also obtained in our study, with patients with dysgeusia having a reduced prevalence of these comorbidities. These findings suggest a possible correlation between the inflammatory response and the pathogenetic mechanisms involved in the development of dysgeusia, a hypothesis that requires further investigation.

With regard to changes in laboratory parameters, the multicenter study by Mahmoud et al. [31] identified an association between dysgeusia and elevated IL-6 and LDH values. It is hypothesized that elevated levels of proinflammatory cytokines may be involved in the pathophysiologic mechanism of dysgeusia, although the exact mechanisms are not fully elucidated. It has been speculated that increased levels of IL-6 may inhibit taste bud turnover [32], while also having an impact on the central nervous system by secondarily affecting the thalamus, the structure where the olfactory and gustatory pathways converge. Furthermore, it could directly influence the activity of astrocytes and microglial cells [33]. However, in our study, neither IL-6 nor LDH were associated with the presence of dysgeusia. A possible explanation for this difference is the higher proportion of patients with moderate and severe forms of COVID-19 in our group, as these forms are generally associated with a heightened inflammatory response, reflected by increased levels of IL-6, IL-1, and LDH. As for lymphopenia, it was present in both groups, but with a higher frequency in patients without dysgeusia. The study by Mobasher et al. [34] showed that lymphopenia is a frequent feature in patients with COVID-19, but did not identify a significant correlation between this change and the presence of taste disorders. In our study, we observed a significantly lower neutrophil-to-lymphocyte ratio (NLR) in patients with dysgeusia compared to those without dysgeusia. Given that NLR is a well-established marker of systemic inflammation and disease severity in COVID-19, this finding suggests that patients experiencing dysgeusia may have had a less pronounced inflammatory response [35]. Conversely, higher NLR levels in patients without dysgeusia may reflect a stronger inflammatory activation, which has been linked to more severe disease outcomes. These findings support the potential role of NLR as a valuable parameter in stratifying COVID-19 severity and warrant further investigation into its relationship with specific clinical manifestations of the disease.

The analysis of other inflammatory markers in our study revealed a significant association between elevated ferritin, fibrinogen, and C-reactive protein values and the absence of dysgeusia. Elevated levels of these markers are known to be predictors of an unfavorable clinical course and are frequently correlated with moderate and severe forms of COVID-19. This finding is consistent with previous data in the literature, which have demonstrated that patients with a marked inflammatory response are at higher risk of severe complications [36,37]. These results suggest that dysgeusia may be associated with a reduced inflammatory response, supporting the hypothesis that this clinical manifestation may have some protective role against severe disease progression. It is important to note that the number of studies investigating the biological profile of patients with dysgeusia in the context of SARS-CoV-2 infection is relatively limited. Therefore, the findings of this study enhance the understanding of the clinical and biological characteristics of these patients and serve as a foundation for future research endeavors.

In terms of the management of patients in our analysis, we identified that individuals experiencing dysgeusia required more intensive and prolonged treatment compared to those who did not report this symptom. This observation suggests that dysgeusia may serve as a clinical marker for patients requiring closer follow-up and potential therapeutic adjustments. Notably, the higher prevalence of dysgeusia among individuals with mild to moderate forms of COVID-19 likely contributed to these findings. Given that dysgeusia is often associated with viral neurotropism and potential inflammatory responses affecting the gustatory pathways, its presence may indicate a broader spectrum of sensory dysfunctions that could influence disease progression and patient recovery trajectories. Furthermore, the persistence of dysgeusia beyond the acute phase of infection underscores the need for targeted therapeutic interventions, including supportive care, nutritional adjustments, and, in some cases, neuromodulatory strategies to facilitate sensory recovery. Additionally, the higher incidence of dysgeusia in patients with mild COVID-19 compared to those with severe disease may reflect differences in symptom reporting rather than a true absence of gustatory dysfunction in critically ill patients. In severe cases, the intense systemic inflammatory response, respiratory distress, and potential neurological complications may overshadow or deprioritize sensory disturbances like dysgeusia.

The majority of previous studies have focused on olfactory disturbances associated with SARS-CoV-2 infection and their impact on clinical course [38,39]. Reported results [40,41,42] showed an association between anosmia and mild forms of COVID-19, which mostly required ambulatory care. In a study conducted in France [42], only 20% of patients with anosmia required care in a specific medical center due to pulmonary involvement, and their clinical course was favorable. It is important to note that most patients with anosmia presented concomitantly with dysgeusia. The results of our study are consistent with these data, indicating that patients with dysgeusia required fewer days of hospitalization and less complex medical care.

Our study has a number of limitations. Firstly, given that most cases of dysgeusia were associated with mild forms of the disease that did not require hospitalization in our clinic, it is possible that the true incidence of dysgeusia in the general population is higher than that reported in this study. The study was also conducted in a single hospital ward, which may limit the generalizability of the results. As a retrospective study, there is a risk that some variables may have been influenced by a lack of objective data or differences in the clinical recording of symptoms. Another limitation of this study is the lack of a severity classification for dysgeusia and the absence of data on food intake and nutritional impact, as these aspects were not systematically recorded in medical charts. However, we believe that this study contributes to the knowledge puzzle on dysgeusia in COVID-19, given the limited number of studies that simultaneously evaluate clinical and laboratory data in patients hospitalized with dysgeusia during COVID-19. The detailed analysis of the medical records of these patients provides valuable insights into the clinical and biological particularities and may contribute to a better understanding of the factors involved in this manifestation. Future studies, prospective and on larger patient samples, are needed to confirm these observations and to fully elucidate the role of dysgeusia in the course of SARS-CoV-2 infection.

## 5. Conclusions

This study showed a 32% incidence of dysgeusia among patients hospitalized for COVID-19, with a notably higher prevalence in those presenting with mild to moderate disease severity. Female sex was strongly correlated with the occurrence of dysgeusia, whereas obesity and high blood hypertension were inversely associated with its presence, without any evidence of a protective effect of them. Moreover, the inflammatory response was diminished in patients exhibiting dysgeusia. Although numerous studies have examined the impact of dysgeusia in the context of SARS-CoV-2 infection, the robustness of these associations remains to be conclusively determined. Therefore, a more detailed characterization of patients with dysgeusia may help to optimize clinical management strategies and more accurately stratify patients diagnosed with COVID-19. In the context of evolving circulating viral variants that appear to be associated with a lower incidence of dysgeusia, continued monitoring of patients who develop this disorder remains essential to elucidate the pathophysiological mechanisms involved and to assess the potential of dysgeusia as a predictor of progression of SARS-CoV-2 infection.

## Figures and Tables

**Table 1:** General characteristics of patients in relation to the presence of dysgeusia.

Feature	All Patients	Patients with Dysgeusia	Patients Without Dysgeusia	*p*-Value
	N = 347	N = 111	N = 236	
**Demographic data**				
Female	189 (54.46%)	73 (65.8%)	116 (49.2%)	0.004
Age, years, median (IQR)	52 (43, 64)	42 (36, 45)	52 (43, 64.5)	0.001
**SARS-CoV-2 variant**				
Alpha	74 (21.3%)	31 (27.9%)	43 (18.2%)	0.077
Beta	179 (51.6%)	49 (44.2%)	130 (55.1%)	
Delta	94 (27.1%)	31 (27.9%)	63 (26.7%)	
**Disease form**				
Mild	53 (15.3%)	38 (34.2%)	15 (6.4%)	0.005
Moderate	106 (30.5%)	40 (36.1%)	66 (27.9%)	
Severe	188 (54.2%)	33 (29.7%)	155 (65.7%)	
**Chronic diseases**				
At least one chronic disease	230 (66.3%)	56 (50.5%)	174 (73.7%)	<0.001
Obesity	164 (47.3%)	38 (34.2%)	126 (53.4%)	0.001
High blood pressure	149 (42.9%)	31 (27.9%)	118 (50.0%)	<0.001
Diabetes mellitus	57 (16.4%)	11 (9.9%)	46 (19.5%)	0.025
Cardiovascular disease	114 (32.8%)	28 (25.2%)	86 (36.4%)	0.038
Chronic lung disease	17 (4.9%)	5 (4.5%)	12 (5.1%)	0.815
Chronic kidney disease	14 (4.0%)	3 (2.7%)	11 (4.7%)	0.387
Neurological disease	17(4.9%)	6 (5.4%)	11 (4.7%)	0.764

IQR—interquartile range.

**Table 2:** Laboratory parameter characteristics according to the presence or absence of dysgeusia.

Lab Parameters		Patients with Dysgeusia N = 111	Patients Without Dysgeusia N = 236	*p*-Value
Neutrophil count	cells/µL median (IQR)	3400 (2500, 4900)	4300 (2900, 6660)	0.325
Neutrophilia	n (%)	16 (14.4%)	56 (23.7%)	0.046
Neutropenia	n (%)	0 (0.0%)	14 (5.9%)	NA
Lymphocyte count	cells/µL median (IQR)	1220 (900, 1900)	885 (600, 1100)	0.002
Lymphocytosis	n (%)	1 (0.9%)	5 (2.1%)	0.417
Lymphopenia	n (%)	51 (45.9%)	187 (79.2%)	<0.001
NLR		2.86 (2.52, 2.97)	4.83 (4.25, 6.03)	<0.001
Fibrinogen	mg/dL median (IQR)	353 (275, 519)	477.5 (372, 577.75)	0.223
High fibrinogen values	n (%)	50 (45.0%)	172 (72.9%)	<0.001
C-reactive protein	mg/L median (IQR)	18 (3, 72)	71 (33, 112.75)	0.111
High C-reactive protein values	n (%)	79 (71.2%)	225 (95.3%)	<0.001
IL-1	pg/L median (IQR)	2 (0.0, 4)	2.5 (0.2, 9)	0.233
High IL-1 values	n (%)	20 (18.0%)	80 (33.9%)	0.002
IL-6	pg/L median (IQR)	31 (2, 140)	50.5 (12.25, 201.5)	0.403
High IL-6 values	n (%)	67 (60.4%)	167 (70.8%)	0.054
LDH	U/L median (IQR)	260 (201, 314)	326.5 (246, 399.75)	0.299
High LDH values	n (%)	62 (55.9%)	177 (75.0%)	<0.001
Feritin	ng/mL median (IQR)	181 (48, 543)	635.5 (269.5, 1329.75)	0.082
High ferritin values	n (%)	43 (38.7%)	165 (69.9%)	<0.001
AST	U/L median (IQR)	43 (27, 48)	59.5 (37, 80)	0.197
High AST values	n (%)	32 (28.8%)	119 (50.4%)	<0.001
ALT	U/L median (IQR)	46 (27, 104)	80.5 (44, 130)	0.325
High ALT values	n (%)	44 (39.6%)	152 (64.4%)	<0.001
Amylase	U/L median (IQR)	52 (39, 78)	71 (49.25, 103.75)	0.560
High amylase values	n (%)	11 (9.9%)	55 (23.3%)	0.003
Lipase	U/L median (IQR)	114 (72, 181)	180 (111, 316.5)	0.138
High lipase values	n (%)	12 (10.8%)	63 (26.7%)	0.001

NA—not applicable; IQR—interquartile range; NLR—neutrophil-to-lymphocyte ratio; IL-1—interleukin 1; IL-6—interleukin 6; AST—aspartate aminotransferase; ALT—alanine aminotransferase.

**Table 3:** Patient management in relation to the presence or absence of dysgeusia.

Type of Treatment	Patients with Dysgeusia N = 111	Patients Without Dysgeusia N = 236	*p*-Value
Antimicrobial therapy, n (%)	84 (75.7%)	216 (91.5%)	<0.001
Antivirals therapy, n (%)	50 (45.0%)	154 (65.3%)	<0.001
Biologic therapy, n (%)	21 (18.9%)	118 (50.0%)	<0.001
Corticosteroids, n (%)	64 (57.7%)	222 (94.1%)	<0.001

**Table 4:** Outcome of patients related to the presence or absence of dysgeusia.

Characteristics	Patients with Dysgeusia N = 111	Patients Without Dysgeusia N = 236	*p*-Value
Days from onset of symptoms to presentation to hospital, median (IQR)	6 (4, 7)	7 (5, 9)	0.345
Length of hospitalization, days, IQR (median)	8 (6, 11)	10 (7, 14)	0.156
Acute respiratory failure, n (%)	32 (28.8%)	168 (71.2%)	<0.001

## Data Availability

The data are available upon reasonable request to the corresponding author.
